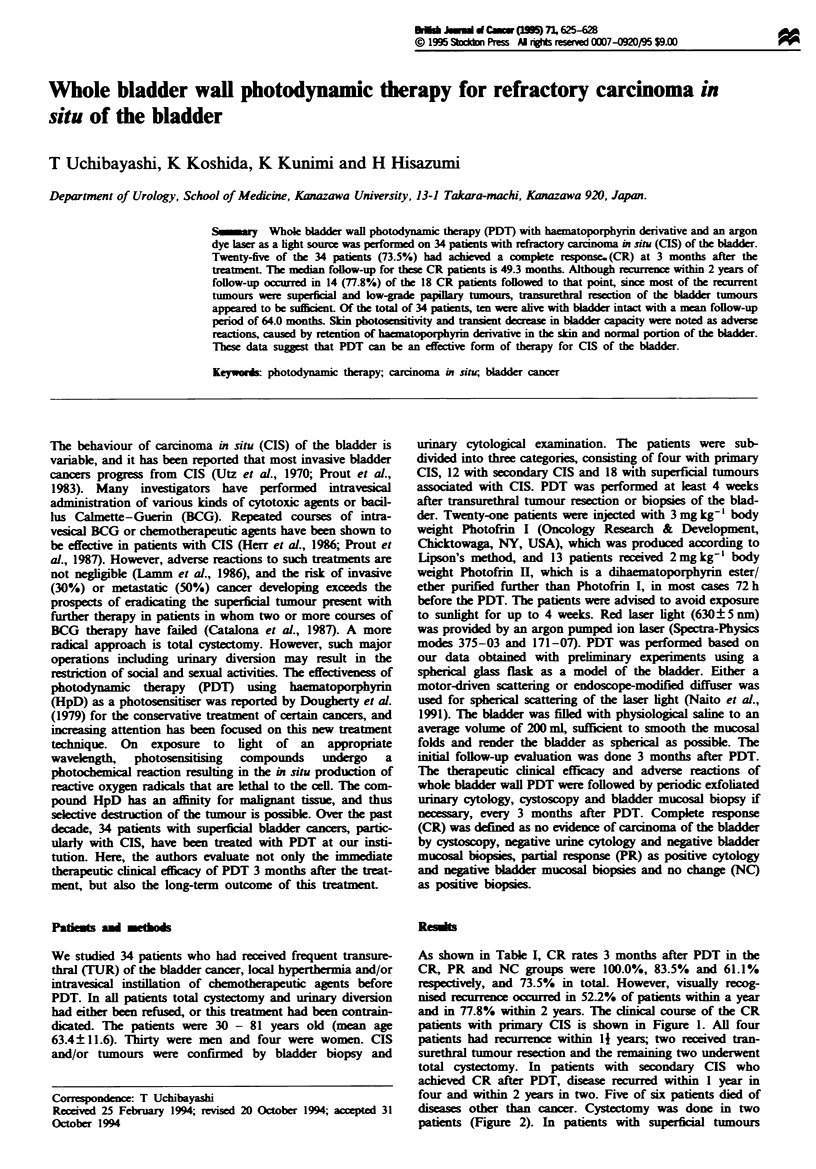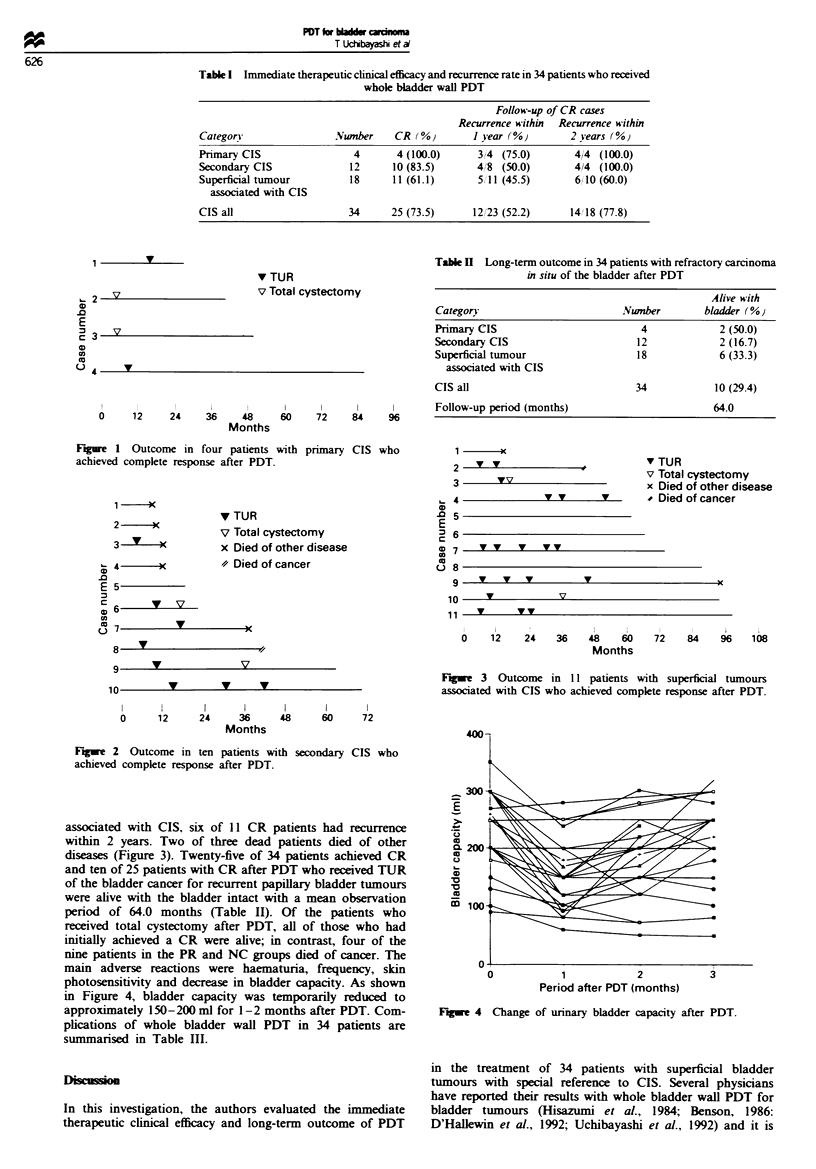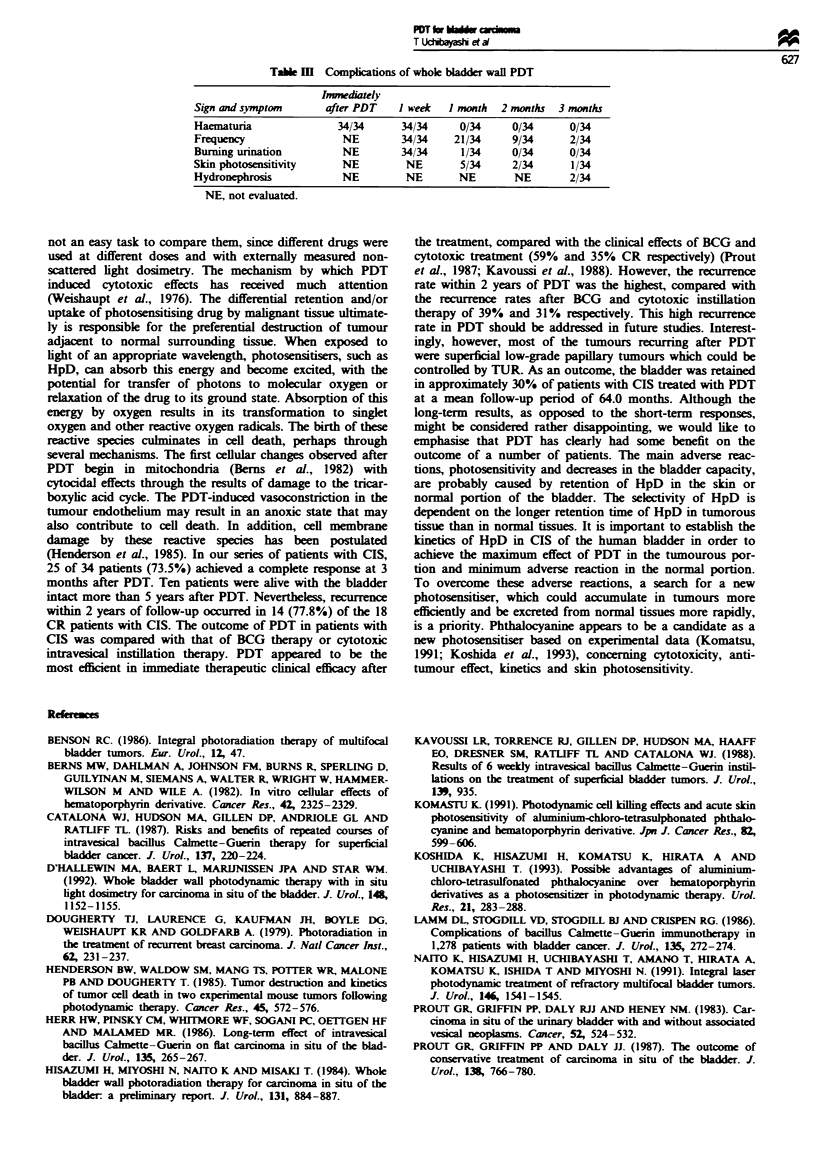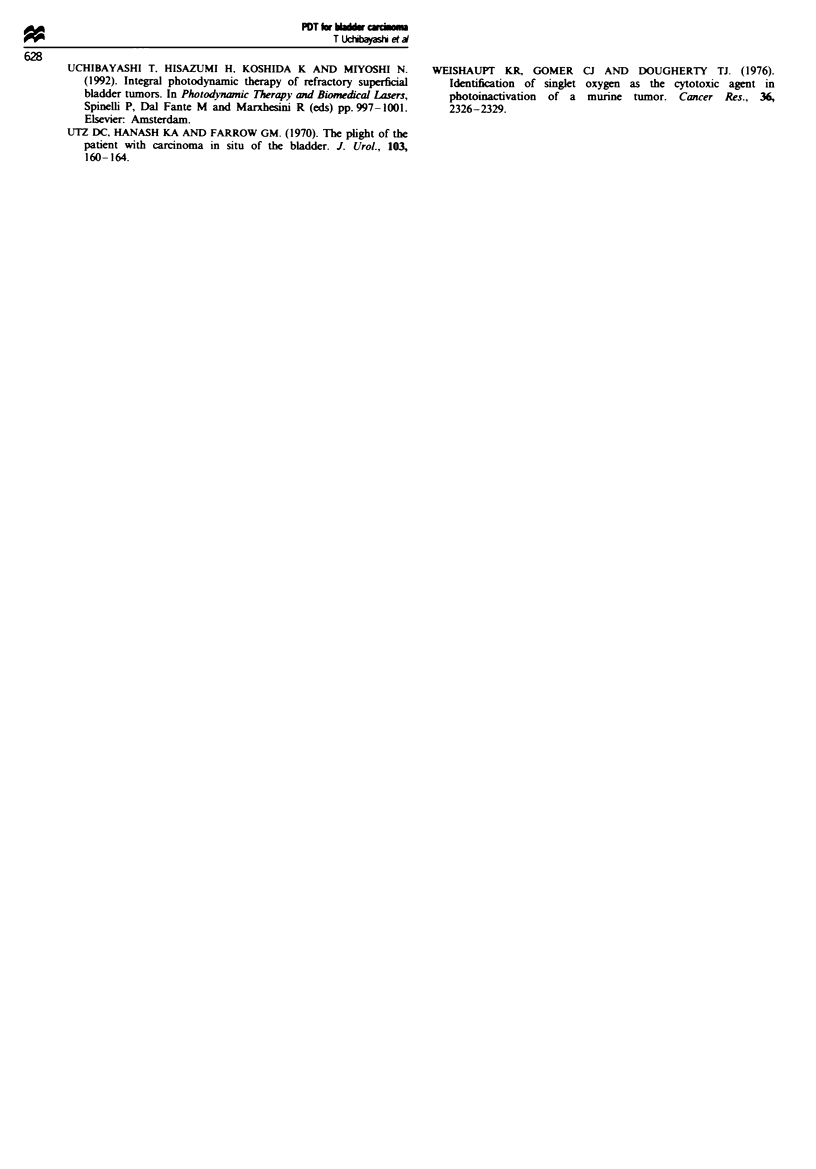# Whole bladder wall photodynamic therapy for refractory carcinoma in situ of the bladder.

**DOI:** 10.1038/bjc.1995.122

**Published:** 1995-03

**Authors:** T. Uchibayashi, K. Koshida, K. Kunimi, H. Hisazumi

**Affiliations:** Department of Urology, School of Medicine, Kanazawa University, Japan.

## Abstract

Whole bladder wall photodynamic therapy (PDT) with haematoporphyrin derivative and an argon dye laser as a light source was performed on 34 patients with refractory carcinoma in situ (CIS) of the bladder. Twenty-five of the 34 patients (73.5%) had achieved a complete response (CR) at 3 months after the treatment. The median follow-up for these CR patients is 49.3 months. Although recurrence within 2 years of follow-up occurred in 14 (77.8%) of the 18 CR patients followed to that point, since most of the recurrent tumours were superficial and low-grade papillary tumours, transurethral resection of the bladder tumours appeared to be sufficient. Of the total of 34 patients, ten were alive with bladder intact with a mean follow-up period of 64.0 months. Skin photosensitivity and transient decrease in bladder capacity were noted as adverse reactions, caused by retention of haematoporphyrin derivative in the skin and normal portion of the bladder. These data suggest that PDT can be an effective form of therapy for CIS of the bladder.


© 1995 Stockton Press All rights reserved 0007-0920/95 $9.00

### Whole bladder wall photodynamic therapy for refractory carcinoma *in situ* of the bladder

T Uchibayashi, K Koshida, K Kunimi and H Hisazumi

*Department of Urology, School of Medicine, Kanazawa University, 13-1 Takara-machi, Kanazawa 920, Japan.*

**Summary** Whole bladder wall photodynamic therapy (PDT) with haematoporphyrin derivative and an argon dye laser as a light source was performed on 34 patients with refractory carcinoma *in situ* (CIS) of the bladder. Twenty-five of the 34 patients (73.5%) had achieved a complete response (CR) at 3 months after the treatment. The median follow-up for these CR patients is 49.3 months. Although recurrence within 2 years of follow-up occurred in 14 (77.8%) of the 18 CR patients followed to that point, since most of the recurrent tumours were superficial and low-grade papillary tumours, transurethral resection of the bladder tumours appeared to be sufficient. Of the total of 34 patients, ten were alive with bladder intact with a mean follow-up period of 64.0 months. Skin photosensitivity and transient decrease in bladder capacity were noted as adverse reactions, caused by retention of haematoporphyrin derivative in the skin and normal portion of the bladder. These data suggest that PDT can be an effective form of therapy for CIS of the bladder.

**Keywords**: photodynamic therapy; carcinoma *in situ*; bladder cancer

The behaviour of carcinoma *in situ* (CIS) of the bladder is variable, and it has been reported that most invasive bladder cancers progress from CIS (Utz *et al.*, 1970; Prout *et al.*, 1983). Many investigators have performed intravesical administration of various kinds of cytotoxic agents or bacillus Calmette-Guerin (BCG). Repeated courses of intravesical BCG or chemotherapeutic agents have been shown to be effective in patients with CIS (Herr *et al.*, 1986; Prout *et al.*, 1987). However, adverse reactions to such treatments are not negligible (Lamm *et al.*, 1986), and the risk of invasive (30%) or metastatic (50%) cancer developing exceeds the prospects of eradicating the superficial tumour present with further therapy in patients in whom two or more courses of BCG therapy have failed (Catalona *et al.*, 1987). A more radical approach is total cystectomy. However, such major operations including urinary diversion may result in the restriction of social and sexual activities. The effectiveness of photodynamic therapy (PDT) using haematoporphyrin (HpD) as a photosensitiser was reported by Dougherty *et al.* (1979) for the conservative treatment of certain cancers, and increasing attention has been focused on this new treatment technique. On exposure to light of an appropriate wavelength, photosensitising compounds undergo a photochemical reaction resulting in the *in situ* production of reactive oxygen radicals that are lethal to the cell. The compound HpD has an affinity for malignant tissue, and thus selective destruction of the tumour is possible. Over the past decade, 34 patients with superficial bladder cancers, particularly with CIS, have been treated with PDT at our institution. Here, the authors evaluate not only the immediate therapeutic clinical efficacy of PDT 3 months after the treatment, but also the long-term outcome of this treatment.

### Patients and methods

We studied 34 patients who had received frequent transurethral (TUR) of the bladder cancer, local hyperthermia and/or intravesical instillation of chemotherapeutic agents before PDT. In all patients total cystectomy and urinary diversion had either been refused, or this treatment had been contraindicated. The patients were 30 - 81 years old (mean age 63.4±11.6). Thirty were men and four were women. CIS and/or tumours were confirmed by bladder biopsy and urinary cytological examination. The patients were subdivided into three categories, consisting of four with primary CIS, 12 with secondary CIS and 18 with superficial tumours associated with CIS. PDT was performed at least 4 weeks after transurethral tumour resection or biopsies of the bladder. Twenty-one patients were injected with 3 mg $kg^{-1}$ body weight Photofrin I (Oncology Research & Development, Chicktowaga, NY, USA), which was produced according to Lipson's method, and 13 patients received 2 mg $kg^{-1}$ body weight Photofrin II, which is a dihaematoporphyrin ester/ether purified further than Photofrin I, in most cases 72 h before the PDT. The patients were advised to avoid exposure to sunlight for up to 4 weeks. Red laser light (630±5 nm) was provided by an argon pumped ion laser (Spectra-Physics modes 375-03 and 171-07). PDT was performed based on our data obtained with preliminary experiments using a spherical glass flask as a model of the bladder. Either a motor-driven scattering or endoscope-modified diffuser was used for spherical scattering of the laser light (Naito *et al.*, 1991). The bladder was filled with physiological saline to an average volume of 200 ml, sufficient to smooth the mucosal folds and render the bladder as spherical as possible. The initial follow-up evaluation was done 3 months after PDT. The therapeutic clinical efficacy and adverse reactions of whole bladder wall PDT were followed by periodic exfoliated urinary cytology, cystoscopy and bladder mucosal biopsy if necessary, every 3 months after PDT. Complete response (CR) was defined as no evidence of carcinoma of the bladder by cystoscopy, negative urine cytology and negative bladder mucosal biopsies, partial response (PR) as positive cytology and negative bladder mucosal biopsies and no change (NC) as positive biopsies.

### Results

As shown in Table I, CR rates 3 months after PDT in the CR, PR and NC groups were 100.0%, 83.5% and 61.1% respectively, and 73.5% in total. However, visually recognised recurrence occurred in 52.2% of patients within a year and in 77.8% within 2 years. The clinical course of the CR patients with primary CIS is shown in Figure 1. All four patients had recurrence within 1½ years; two received transurethral tumour resection and the remaining two underwent total cystectomy. In patients with secondary CIS who achieved CR after PDT, disease recurred within 1 year in four and within 2 years in two. Five of six patients died of diseases other than cancer. Cystectomy was done in two patients (Figure 2). In patients with superficial tumours

Correspondence: T Uchibayashi
Received 25 February 1994; revised 20 October 1994; accepted 31 October 1994

**Table I** Immediate therapeutic clinical efficacy and recurrence rate in 34 patients who received whole bladder wall PDT

| Category | Number | CR (%) | Follow-up of CR cases: Recurrence within 1 year (%) | Follow-up of CR cases: Recurrence within 2 years (%) |
|---|---|---|---|---|
| Primary CIS | 4 | 4 (100.0) | 3/4 (75.0) | 4/4 (100.0) |
| Secondary CIS | 12 | 10 (83.5) | 4/8 (50.0) | 4/4 (100.0) |
| Superficial tumour associated with CIS | 18 | 11 (61.1) | 5/11 (45.5) | 6/10 (60.0) |
| CIS all | 34 | 25 (73.5) | 12/23 (52.2) | 14/18 (77.8) |

**Figure 1** Outcome in four patients with primary CIS who achieved complete response after PDT.

**Figure 2** Outcome in ten patients with secondary CIS who achieved complete response after PDT.

**Table II** Long-term outcome in 34 patients with refractory carcinoma *in situ* of the bladder after PDT

| Category | Number | Alive with bladder (%) |
|---|---|---|
| Primary CIS | 4 | 2 (50.0) |
| Secondary CIS | 12 | 2 (16.7) |
| Superficial tumour associated with CIS | 18 | 6 (33.3) |
| CIS all | 34 | 10 (29.4) |
| Follow-up period (months) | | 64.0 |

**Figure 3** Outcome in 11 patients with superficial tumours associated with CIS who achieved complete response after PDT.

**Figure 4** Change of urinary bladder capacity after PDT.

associated with CIS, six of 11 CR patients had recurrence within 2 years. Two of three dead patients died of other diseases (Figure 3). Twenty-five of 34 patients achieved CR and ten of 25 patients with CR after PDT who received TUR of the bladder cancer for recurrent papillary bladder tumours were alive with the bladder intact with a mean observation period of 64.0 months (Table II). Of the patients who received total cystectomy after PDT, all of those who had initially achieved a CR were alive; in contrast, four of the nine patients in the PR and NC groups died of cancer. The main adverse reactions were haematuria, frequency, skin photosensitivity and decrease in bladder capacity. As shown in Figure 4, bladder capacity was temporarily reduced to approximately 150–200 ml for 1–2 months after PDT. Complications of whole bladder wall PDT in 34 patients are summarised in Table III.

### Discussion

In this investigation, the authors evaluated the immediate therapeutic clinical efficacy and long-term outcome of PDT in the treatment of 34 patients with superficial bladder tumours with special reference to CIS. Several physicians have reported their results with whole bladder wall PDT for bladder tumours (Hisazumi *et al.*, 1984; Benson, 1986: D'Hallewin *et al.*, 1992; Uchibayashi *et al.*, 1992) and it is

Table III Complications of whole bladder wall PDT

| Sign and symptom | Immediately after PDT | 1 week | 1 month | 2 months | 3 months |
|---|---|---|---|---|---|
| Haematuria | 34/34 | 34/34 | 0/34 | 0/34 | 0/34 |
| Frequency | NE | 34/34 | 21/34 | 9/34 | 2/34 |
| Burning urination | NE | 34/34 | 1/34 | 0/34 | 0/34 |
| Skin photosensitivity | NE | NE | 5/34 | 2/34 | 1/34 |
| Hydronephrosis | NE | NE | NE | NE | 2/34 |

NE, not evaluated.

not an easy task to compare them, since different drugs were used at different doses and with externally measured non-scattered light dosimetry. The mechanism by which PDT induced cytotoxic effects has received much attention (Weishaupt *et al.*, 1976). The differential retention and/or uptake of photosensitising drug by malignant tissue ultimately is responsible for the preferential destruction of tumour adjacent to normal surrounding tissue. When exposed to light of an appropriate wavelength, photosensitisers, such as HpD, can absorb this energy and become excited, with the potential for transfer of photons to molecular oxygen or relaxation of the drug to its ground state. Absorption of this energy by oxygen results in its transformation to singlet oxygen and other reactive oxygen radicals. The birth of these reactive species culminates in cell death, perhaps through several mechanisms. The first cellular changes observed after PDT begin in mitochondria (Berns *et al.*, 1982) with cytocidal effects through the results of damage to the tricarboxylic acid cycle. The PDT-induced vasoconstriction in the tumour endothelium may result in an anoxic state that may also contribute to cell death. In addition, cell membrane damage by these reactive species has been postulated (Henderson *et al.*, 1985). In our series of patients with CIS, 25 of 34 patients (73.5%) achieved a complete response at 3 months after PDT. Ten patients were alive with the bladder intact more than 5 years after PDT. Nevertheless, recurrence within 2 years of follow-up occurred in 14 (77.8%) of the 18 CR patients with CIS. The outcome of PDT in patients with CIS was compared with that of BCG therapy or cytotoxic intravesical instillation therapy. PDT appeared to be the most efficient in immediate therapeutic clinical efficacy after the treatment, compared with the clinical effects of BCG and cytotoxic treatment (59% and 35% CR respectively) (Prout *et al.*, 1987; Kavoussi *et al.*, 1988). However, the recurrence rate within 2 years of PDT was the highest, compared with the recurrence rates after BCG and cytotoxic instillation therapy of 39% and 31% respectively. This high recurrence rate in PDT should be addressed in future studies. Interestingly, however, most of the tumours recurring after PDT were superficial low-grade papillary tumours which could be controlled by TUR. As an outcome, the bladder was retained in approximately 30% of patients with CIS treated with PDT at a mean follow-up period of 64.0 months. Although the long-term results, as opposed to the short-term responses, might be considered rather disappointing, we would like to emphasise that PDT has clearly had some benefit on the outcome of a number of patients. The main adverse reactions, photosensitivity and decreases in the bladder capacity, are probably caused by retention of HpD in the skin or normal portion of the bladder. The selectivity of HpD is dependent on the longer retention time of HpD in tumorous tissue than in normal tissues. It is important to establish the kinetics of HpD in CIS of the human bladder in order to achieve the maximum effect of PDT in the tumourous portion and minimum adverse reaction in the normal portion. To overcome these adverse reactions, a search for a new photosensitiser, which could accumulate in tumours more efficiently and be excreted from normal tissues more rapidly, is a priority. Phthalocyanine appears to be a candidate as a new photosensitiser based on experimental data (Komatsu, 1991; Koshida *et al.*, 1993), concerning cytotoxicity, antitumour effect, kinetics and skin photosensitivity.

### References

BENSON RC. (1986). Integral photoradiation therapy of multifocal bladder tumors. *Eur. Urol.*, **12**, 47.

BERNS MW, DAHLMAN A, JOHNSON FM, BURNS R, SPERLING D, GUILYINAN M, SIEMANS A, WALTER R, WRIGHT W, HAMMER-WILSON M AND WILE A. (1982). In vitro cellular effects of hematoporphyrin derivative. *Cancer Res.*, **42**, 2325-2329.

CATALONA WJ, HUDSON MA, GILLEN DP, ANDRIOLE GL AND RATLIFF TL. (1987). Risks and benefits of repeated courses of intravesical bacillus Calmette-Guerin therapy for superficial bladder cancer. *J. Urol.*, **137**, 220-224.

D'HALLEWIN MA, BAERT L, MARIJNISSEN JPA AND STAR WM. (1992). Whole bladder wall photodynamic therapy with in situ light dosimetry for carcinoma in situ of the bladder. *J. Urol.*, **148**, 1152-1155.

DOUGHERTY TJ, LAURENCE G, KAUFMAN JH, BOYLE DG, WEISHAUPT KR AND GOLDFARB A. (1979). Photoradiation in the treatment of recurrent breast carcinoma. *J. Natl Cancer Inst.*, **62**, 231-237.

HENDERSON BW, WALDOW SM, MANG TS, POTTER WR, MALONE PB AND DOUGHERTY T. (1985). Tumor destruction and kinetics of tumor cell death in two experimental mouse tumors following photodynamic therapy. *Cancer Res.*, **45**, 572-576.

HERR HW, PINSKY CM, WHITMORE WF, SOGANI PC, OETTGEN HF AND MALAMED MR. (1986). Long-term effect of intravesical bacillus Calmette-Guerin on flat carcinoma in situ of the bladder. *J. Urol.*, **135**, 265-267.

HISAZUMI H, MIYOSHI N, NAITO K AND MISAKI T. (1984). Whole bladder wall photoradiation therapy for carcinoma in situ of the bladder: a preliminary report. *J. Urol.*, **131**, 884-887.

KAVOUSSI LR, TORRENCE RJ, GILLEN DP, HUDSON MA, HAAFF EO, DRESNER SM, RATLIFF TL AND CATALONA WJ. (1988). Results of 6 weekly intravesical bacillus Calmette-Guerin instillations on the treatment of superficial bladder tumors. *J. Urol.*, **139**, 935.

KOMASTU K. (1991). Photodynamic cell killing effects and acute skin photosensitivity of aluminium-chloro-tetrasulphonated phthalocyanine and hematoporphyrin derivative. *Jpn J. Cancer Res.*, **82**, 599-606.

KOSHIDA K, HISAZUMI H, KOMATSU K, HIRATA A AND UCHIBAYASHI T. (1993). Possible advantages of aluminium-chloro-tetrasulfonated phthalocyanine over hematoporphyrin derivatives as a photosensitizer in photodynamic therapy. *Urol. Res.*, **21**, 283-288.

LAMM DL, STOGDILL VD, STOGDILL BJ AND CRISPEN RG. (1986). Complications of bacillus Calmette-Guerin immunotherapy in 1,278 patients with bladder cancer. *J. Urol.*, **135**, 272-274.

NAITO K, HISAZUMI H, UCHIBAYASHI T, AMANO T, HIRATA A, KOMATSU K, ISHIDA T AND MIYOSHI N. (1991). Integral laser photodynamic treatment of refractory multifocal bladder tumors. *J. Urol.*, **146**, 1541-1545.

PROUT GR, GRIFFIN PP, DALY RJJ AND HENEY NM. (1983). Carcinoma in situ of the urinary bladder with and without associated vesical neoplasms. *Cancer*, **52**, 524-532.

PROUT GR, GRIFFIN PP AND DALY JJ. (1987). The outcome of conservative treatment of carcinoma in situ of the bladder. *J. Urol.*, **138**, 766-780.

UCHIBAYASHI T, HISAZUMI H, KOSHIDA K AND MIYOSHI N. (1992). Integral photodynamic therapy of refractory superficial bladder tumors. In *Photodynamic Therapy and Biomedical Lasers*, Spinelli P, Dal Fante M and Marxhesini R (eds) pp. 997-1001. Elsevier: Amsterdam.

UTZ DC, HANASH KA AND FARROW GM. (1970). The plight of the patient with carcinoma in situ of the bladder. *J. Urol.*, **103**, 160-164.

WEISHAUPT KR, GOMER CJ AND DOUGHERTY TJ. (1976). Identification of singlet oxygen as the cytotoxic agent in photoinactivation of a murine tumor. *Cancer Res.*, **36**, 2326-2329.